# Psychological resilience and mood disorders: a systematic review and meta-analysis

**DOI:** 10.47626/2237-6089-2022-0524

**Published:** 2024-02-28

**Authors:** Areeba Imran, Suleman Tariq, Flavio Kapczinski, Taiane de Azevedo Cardoso

**Affiliations:** 1 Life Sciences Program School of Interdisciplinary Science McMaster University Hamilton ON Canada Life Sciences Program, School of Interdisciplinary Science, McMaster University, Hamilton, ON, Canada.; 2 Health Sciences Program Faculty of Health Sciences McMaster University Hamilton ON Canada Health Sciences Program, Faculty of Health Sciences, McMaster University, Hamilton, ON, Canada.; 3 Department of Psychiatry and Behavioural Neurosciences McMaster University Hamilton ON Canada Department of Psychiatry and Behavioural Neurosciences, McMaster University, Hamilton, ON, Canada.; 4 Instituto Nacional de Ciência e Tecnologia Translacional em Medicina Porto Alegre RS Brazil Instituto Nacional de Ciência e Tecnologia Translacional em Medicina (INCT-TM), Porto Alegre, RS, Brazil.; 5 Laboratório de Psiquiatria Molecular Hospital de Clínicas de Porto Alegre Porto Alegre RS Brazil Laboratório de Psiquiatria Molecular, Hospital de Clínicas de Porto Alegre, Porto Alegre, RS, Brazil.; 6 Departamento de Psiquiatria Universidade Federal do Rio Grande do Sul Porto Alegre RS Brazil Departamento de Psiquiatria, Universidade Federal do Rio Grande do Sul, Porto Alegre, RS, Brazil.

**Keywords:** Mood disorders, psychological resilience, bipolar disorder, depression, systematic review, meta-analysis

## Abstract

**Objective:**

This systematic review aims to describe the relationship between psychological resilience and mood disorders.

**Methods:**

This is a systematic review and meta-analysis. The following databases were searched on November 6, 2020: PubMed, PsycINFO, and Embase.

**Results:**

Twenty-three articles were included and the majority of the studies included (95.7%) showed that psychological resilience has a positive impact in mood disorders. Our meta-analysis showed that individuals with bipolar disorder presented significantly lower levels of psychological resilience compared to controls (standardized mean difference [SDM]: -0.99 [95% confidence interval {95%CI}: -1.13 to -0.85], p < 0.001). In addition, individuals with depression had significantly lower levels of psychological resilience compared to controls (SDM: -0.71 [95%CI -0.81 to -0.61], p < 0.001).

**Conclusion:**

Our results showed that individuals with mood disorders are less resilient than individuals without mood disorders. Our findings reinforce the importance of investigating interventions that may help to improve psychological resilience considering its positive impact in the context of mood disorders.

## Introduction

Mood disorders have high prevalence worldwide and are associated with increased rates of disability. The lifetime prevalence of major depressive disorder (MDD) in high-income countries is 14.6%^1^ and the prevalence in low-to-middle-income countries is 11.1%,^1^ while the lifetime prevalence of bipolar disorder (BD) worldwide is 2.4%.^2^ Mood disorders are associated with reduced quality of life (QoL),^3^ increased functional impairment,^4^ and increased suicide risk,^5^ even in a young adult population. Importantly, in a large population-based cohort study published in 2020, Frey et al.^6^ showed that mood disorders were associated with elevated and early rates of receiving disability services. These data reinforce the negative impact of mood disorders on individuals’ lives. Hence, it is necessary to evaluate strategies that can potentially limit this negative impact.

Current literature suggests a relationship between childhood adversity and mood disorders. Being a victim of bullying and emotional abuse or emotional neglect during childhood have been shown to be strong predictors of depression.^7^ Importantly, a recent study showed that resilience partly mediated the association of childhood trauma with both mood disorders and severity of depression, meaning that individuals who suffered from trauma but were more resilient were less likely to develop mood disorders.^8^ This reinforces the importance of studying resilience in the context of mood disorders.

Resilience is a complex multidimensional construct defined as the ability to adapt successfully in the face of stress and adversity, maintaining normal psychological and physical functioning.^9^ According to the American Association of Psychology (APA), psychological resilience is the ability to be able to “bounce back” from stressful times.^10^ Currently, to the best of our knowledge, there are only two systematic reviews that have assessed the relationship between mental health and resilience. In 2014, Siriwardhana et al.^11^ examined the relationship between mental health and resilience in adults who were forced to migrate and showed a positive impact of resilience on the mental health of these individuals. In 2018, Färber et al.^12^ examined the relationship between mental health and resilience in somatically ill adults and concluded that higher resilience led to better mental health when participants were suffering from a physical illness. It is important to point out that these reviews were focused on specific populations (individuals forced to migrate and individuals with somatic illness) and they did not specifically assess the impact of psychological resilience on mood disorders.

Thus, the aim of our systematic review is to describe the impact of psychological resilience in mood disorders.

## Methods

The Preferred Reporting Items for Systematic Reviews and Meta-Analysis (PRISMA) guidelines were followed for the present review.

### Protocol registration

A protocol for this systematic review was registered prospectively with PROSPERO on November 23, 2020, under ID CRD42020214767.

### Search strategy

A literature search with no year or language restrictions was conducted on November 6, 2020, using the following databases: PubMed, PsycINFO, and Embase. We searched for a combination of the following search items (“mood disorder” OR “mood disorders” OR “depression” OR “major depression” OR “major depressive disorder” OR “depressive episode” OR “dysthymia” OR “bipolar disorder” OR “bipolar disorders” OR “mania” OR “manic” OR “hypomanic”) AND (“resilience” OR “Psychological Resilience” OR “Psychological Resiliences”). The search yielded 15,749 articles (PubMed = 5,052, PsycINFO = 4,783, and Embase = 5,914), with 9,903 remaining after removal of duplicate (5,846 removed).

We used the following inclusion criteria to determine whether an article was relevant to our study: (1) the study should present original data; (2) cross-sectional studies should include individuals with depression or BD and compare their levels of resilience with individuals without depression or BD; and (3) prospective cohort studies and clinical trials should include individuals with depression or BD and assess the effect of resilience on mood symptoms over time. The exclusion criteria were (1) reviews and meta-analyses, (2) case reports or case series, and (3) conference abstracts.

**Figure 1:**
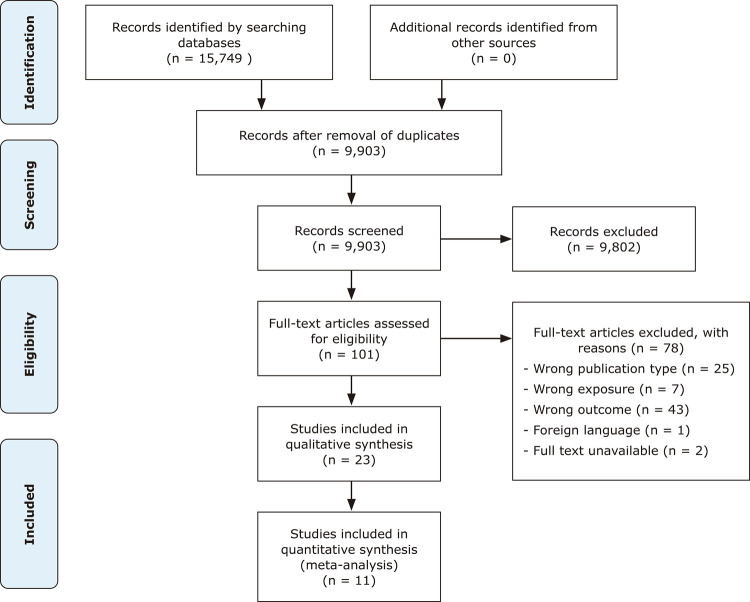
Preferred Reporting Items for Systematic Reviews and Meta-Analysis (PRISMA) 2009 flow diagram.

The studies were assessed by two blinded raters (ST and AI), who determined whether the studies met the inclusion criteria. Each rater assessed manuscripts independently using the Rayyan platform and divergences were resolved in a meeting with a third researcher (TAC). The raters first screened articles by title and abstract and then by full text. All articles not fulfilling the search criteria were excluded. The details of the search strategy are illustrated in Figure 1.

### Data extraction

Two researchers (ST and AI) conducted the data extraction process. They extracted authorship, year of publication, the country in which the study took place, study aims, characteristics of the population, confounding variables controlled, assessments, and main results.

### Quality assessment

All 23 studies included were independently assessed by two blind researchers (ST and AI) using the Joanna Briggs Institute (JBI) critical appraisal tools. Disagreements were resolved during a meeting with a third researcher (TAC).

### Statistical analysis

Meta-analyses were conducted using Review Manager 5.4 software. Random effects analyses were performed to compare psychological resilience scores between individuals with BD and controls and between individuals with depression and controls. For this purpose, the reported means, sample sizes, and standard deviation (SD) were used to compute standardized mean difference (SDM) between the groups. Significance was set at p < 0.05. Cochrane’s Q test was performed to assess statistical heterogeneity and the Higgins I^2^ statistic was used to determine the extent of variation between sample estimates, with values ranging from 0 to 100%. If information was not reported in the paper, we contacted the authors asking for additional information in order to include their paper in the meta-analysis.

## Results

The literature search resulted in 15,749 articles from the three databases PubMed (5,052), PsycINFO (4,783), and Embase (5,914). Of these, 5,846 were duplicates, and 9,802 studies were excluded because the titles and abstracts were not relevant to the research topic, leaving 101 potentially eligible studies for full-text screening. After this stage, a further 78 studies did not meet the inclusion criteria and a total of 23 studies were included in the systematic review.

The characteristics of the studies included are described in Table 1. The publication dates ranged from 2000 to 2020. The studies were conducted in many different countries, as follows: the United States (n = 5), China (n = 3), South Korea (n = 3), Brazil (n = 2), Turkey (n = 2), Taiwan (n = 1), Russia (n = 1), Japan (n =1), Austria (n = 1), Greece (n = 1), Sweden (n = 1), Belgium (n = 1), and Scotland (n = 1). The studies had total sample sizes ranging from 52 to 213,693 individuals. All studies included individuals with mood disorders (depression and/or BD) and assessed psychological resilience. The Connor-Davidson Resilience Scale (CD-RISC) was the most common assessment instrument used to measure psychological resilience. MDD was most commonly assessed using the Diagnostic and Statistical Manual of Mental Disorders, 4th edition (DSM-IV). BD was most commonly assessed using the ICD criteria. Seventeen studies had a cross-sectional design, four studies had a longitudinal study design, and two were interventional studies.

**Table 1:** Characteristics of the studies included

Author, year, country	Aim	Sample characteristics	Assessments	Confounding factors controlled	Main results	Quality	Are individuals with mood disorders less resilient than the controls?	
Cross-sectional studies								
Zhang et al.,^13^ 2020, China	To assess the prevalence of prenatal depression and explore its associated factors	605 pregnant women from three hospitals in two provincial capitals (Shenyang and Zhengzhou) and one municipality (Chongqing) were included. The maximum age was 35. 433 women had no prenatal depression. 172 had prenatal depression.	A smartphone CES-D questionnaire was used to assess prenatal depression. Resilience was measured using the 14-item Ego RS.	N/A	Individuals with prenatal depression had a higher likelihood (75%) of being in the group with lower resilience scores (80 or less) compared to individuals without prenatal depression (40.9%, p < 0.001).	6/7	Yes	
Elmore et al.,^14^ 2020, United States	To examine the associations between exposure to adverse childhood experiences and positive childhood experiences and depression	Non-institutionalized households with at least one child between 0 and 17 in the United States were randomly selected for the survey. If the parent or caregiver had more than one child, the interviewer randomly chose a single child for the interview. These children were 8 years or older. The final sample was 40,302 children. The sample was divided between currently depressed (n = 2,174) and not currently depressed (38,128).	Resilience and depression were measured using the NSCH survey. Depression was self-reported. In order to be a part of the depression group, parents had to answer yes to the following questions: “Has a doctor ever told you this child has...” for 26 independent health conditions. If they answered yes, a secondary question, “If yes, does this child CURRENTLY have this condition?” was answered.	Race, age, relation to the child, insurance, adult education, special healthcare needs, and caregiver mental health.	Children who were currently depressed were less likely to report child resilience. The presence of child resilience reduced the odds of depression fourfold. Unadjusted OR: 8.17 (manually calculated using data from Table 2); adjusted OR (95%CI); 3.74 (2.88-4.84).	5/7	Yes	
Seok et al.,^15^ 2012, South Korea	To assess the relationships between depressive symptoms, early-life stress, and resilience in MDD	26 patients with MDD (seven males and 19 females; mean age of 31.9 ± 1.8 years) were recruited by hospital staff psychiatrists. 26 age and gender-matched healthy controls (mean age of 32.3 ± 1.7 years) were recruited from the community.	Diagnosis of MDD was confirmed using the Korean version of the SCID for DSM-IV. Resilience was measured using the CD-RISC. Resilience was split into five factors.	The control group and the group with MDD were matched based on gender and age.	Controls had higher resilience scores than individuals with MDD. Resilience was divided into the factors below: Self-efficacy MDD: 12.8 ± 1.4 Control: 17.1 ± 1.1 T-score: -2.358 p-value: 0.022 Self-confidence MDD: 11.8 ± 1.3 Control: 20.0 ± 0.8 T-score: -5.258 P-value: < 0.001 Optimism MDD: 8.2 ± 0.7 Control: 10.9 ± 0.6 T-score: -2.860 p-value: 0.006 Self-control MDD: 5.6 ± 0.7 Control: 9.7 ± 0.5 T-score: -4.502 p-value: < 0.001 Spirituality/autonomy MDD: 5.5 ± 0.7 Control: 7.2 ± 0.5 T-score: -2.506 p-value: 0.041	7/7	Yes	
Cha et al.,^16^ 2014, South Korea	To investigate the demographic and clinical factors related to resilience in euthymic patients with BD. The association between impulsivity and resilience was also investigated.	A total of 62 outpatients with BD type I, II, and NOS who were in remission were recruited along with 62 healthy individuals matched to the BD group for age and sex.	Cases were diagnosed in accordance with the DSM-IV-TR criteria. Resilience was measured using the CD-RISC.	Length of education and employment status. The control group and the group with BD were matched for age and sex.	The resilience scores were higher in controls (72.77 ± 10.14) than in individuals with BD (60.58 ± 18.89, p < 0.001). The results remained significant after adjusting for confounders.	7/7	Yes	
Ozawa et al.,^17^ 2017, Japan	To address the degree and quality of resilience in patients with depression in the context of remission status, spirituality/religiosity, and family members’ resilience levels	The sample was collected from ten psychiatric hospitals and clinics in Tokyo and Saitama, Japan. The sample comprised outpatients 18 years and older. The control group were family members with no depression. There were 36 people in the control group. 100 outpatients with depression were examined.	Depression was diagnosed with ICD-10. Resilience was measured using the 25-item RS.	There were no significant differences between the control group and the group with depression in terms of years of education.	The RS total score was higher in controls (118.9 ± 22.0) than in individuals with depression (100.8 ± 25.9, p < 0.001).	6/7	Yes	
Deng et al.,^18^ 2018, China	To examine the relationship between resilience and cognitive function in patients with schizophrenia, patients with BD, and healthy controls	81 patients with schizophrenia and 34 with BD were recruited from the inpatient and outpatient units of the Department of Psychiatry of the Second Xiangya Hospital of Central South University, Changsha, China. 52 people were in the healthy control group.	BD was diagnosed using the SCID for DSM-IV. Resilience was measured using the CD-RISC (Chinese version).	Years of education, gender, marital status, and employment. The control group, the group with BD, and the group with schizophrenia were matched for age	The resilience scores were higher in controls (69.83 ± 11.70) than in individuals with BD (61.44 ± 18.1, p < 0.02). The difference between schizophrenia, BD, and controls remained significant after adjusting for confounders.	7/7	Yes	
Bozikas et al.,^19^ 2018, Greece	To examine the association between resilience and social functioning in patients with BD	40 clinically stable patients with BD type I and BD type II were included. 40 healthy controls matched for age, sex, and educational background were also included.	BD diagnosis was completed using the DSM-IV and diagnosis was confirmed using the Greek version of the MINI. Resilience was measured using the CD-RISC.	The control group and the group with BD were matched for age, sex, and educational background.	The resilience scores were higher in controls (73.25 ± 9.12) than in individuals with BD (61.98 ± 12.811, p < 0.001).	7/7	Yes	
Post et al.,^20^ 2018, Austria	To examine to what extent resilience, internalized stigma, and psychopathology are correlated with QoL	60 outpatients diagnosed with BD-I and 77 healthy control subjects from the general community were included.	BD was diagnosed in accordance with the DSM-IV criteria. Resilience was measured using the 25-item RS.	There were no significant differences between the control group and the group with BD in terms of years of education or age.	The resilience scores were higher in controls (150.4 ± 14) than in individuals with BD (129.8 ± 2, p < 0.001).	7/7	Yes	
Vieira et al.,^8^ 2020, Brazil	To assess the mediation effect of resilience on the relationship between childhood trauma and mood disorders, as well as the severity of depressive symptoms in a population-based sample	There were 837 individuals in the control group. There were 317 individuals in the MDD group. There were 90 individuals in the BD group.	Mood disorders were assessed using the MINI- PLUS. The severity of depressive symptoms was assessed using the MADRS scale. Resilience was measured using the 25-item RS.	N/A	The resilience scores were higher in controls (139.61 ± 17.60) than in individuals with MDD (129.95 ± 22.72) and BD (122.30 ± 24.77, p < 0.001).	5/7	Yes	
Uygun et al.,^21^ 2020, Turkey	To examine the relationship between perceived social support and resilience in individuals with BD	90 euthymic individuals with BD and 30 controls were included. Age ranged from 18-65 years.	Patients had already been diagnosed with BD prior to the study. Resilience was measured using the Psychological RSA.	The control group and the group with BD were matched for age, gender, marital status, and educational level.	The resilience scores were higher in controls (111.2 ± 4.43) than in individuals with BD (98.91 ± 17.89, p = 0.0001).	7/7	Yes	
Aroian et al.,^22^ 2000, Russia	To assess the relationships between resilience, demographic characteristics, immigration demands, and depression in a sample of 450 adult Russian immigrants to Israel	450 Russian immigrants who emigrated from the former Soviet Union to Northern Israel between 1990 and 1995. 241 people had depression. 209 did not have depression.	Depression was measured using the 13-item Depression Scale of the SCL-90-R (Russian Version). Resilience was measured using the RS developed by Wagnild and Youngís (1993).	N/A	The odds of not being depressed given an increase in resilience were increased about twofold (p = 0.0001).	6/7	Yes	
Hsieh et al.,^23^ 2016, Taiwan	To examine the relationships among recent workplace violence, depressive tendency, social support, and resilience in victimized nurses	The sample was recruited from two hospitals in Taiwan. 159 nurses met the inclusion criteria and were divided between a group with a depressive tendency (n = 74) and a group without depressive tendencies (n = 85).	Depressive tendency was measured using the CES-D. The cut-off used for depressive tendency was 14. Resilience was measured using the RS developed by Friborget al. (2006).	There were no significant differences between the group without a depressive tendency and the group with a depressive tendency in terms of education or age.	The group without a depressive tendency had higher resilience scores (157.94 ± 26.30) compared to the group with a depressive tendency (135.19 ± 15.66, p < 0.001).	4/7	Yes	
Blackmon et al.,^24^ 2017, United States	To examine relationships between depression, psychological resilience, and other sociodemographic factors in individuals highly exposed to Hurricane Katrina in 2005 and the Deepwater Horizon Oil Spill in 2010	A spatially stratified random sample of 292 Mississippi Gulf Coast residents living close to the Gulf of Mexico was assessed. 61 people (21%) had depression. 231 people did not have depression.	Depression was measured using the CES-D. A cut-off of 16 was used. Resilience was measured using the self-rated measure from the 10-item CD-RISC.	Education (less than high school vs. bachelor’s degree or higher), health insurance, Katrina-related damages, and oil spill-related damages. There were no significant differences between the group without depression and the group with depression in terms of gender.	Individuals without depression had higher resilience scores (33.70 ± 6.2) than individuals with depression (27.30 ± 8.08, p < 0.001).	6/7	Yes	
Simpkin et al.,^25^ 2018, United States	To determine how stress from uncertainty is related to resilience among pediatric residents and whether these attributes are associated with depression and burnout	50 residents were surveyed from pediatric residency programs at four urban freestanding children’s hospitals in North America in 2015. 5 residents fulfilled the criteria for depression. 45 were not depressed.	Depression was measured using the HANDS. Resilience was measured using the 14-item RS.	N/A	Individuals without depression had higher resilience scores (85.4 ± 8.0) than individuals with depression (56.6 ± 10.7, p < 0.001).	4/7	Yes	
Poudel-Tandukar et al.,^26^ 2019, United States	To assess the association between resilience and anxiety or depression in Bhutanese adults resettled in Western Massachusetts	450 Bhutanese refugees aged 20-65 residing in Massachusetts were included. 54 had depression. 171 did not have depression.	The HSC-25 was used to measure anxiety (10-items) and depression (15 items) with a cutoff mean score of ≥ 1.75 for moderate to severe symptoms. Resilience was measured using the 25-item Wagnild and Young RS.	Age, sex, marital status, education, occupation, time living in the United States, alcohol intake, smoking, physical activity, history of any chronic disease, coping style, and social support.	Participants in the highest tertile by resilience scores had a significantly decreased risk of depression (OR: 0.16 [95%CI 0.04-0.60], p = 0.010).	6/7	Yes	
Yörük et al.,^27^ 2020, Turkey	To determine the relationship between psychological resilience, burnout, stress, and sociodemographic factors and depression in nurses and midwives during the COVID-19 pandemic	377 midwives and nurses were included (120 with depression and 257 without depression).	Depression was measured using the BDI. The cut-off for depression was 17. Resilience was measured using the RS for adults developed by Friborg et al.	N/A	The group without depression had higher resilience scores (129.78 ± 17.85) than the group with depression (114.35 ± 14.95, p < 0.001).	4/7	Yes	
Barzilay et al.,^28^ 2020, United States	To assess the role of resilience for healthcare workers during the COVID-19 pandemic	This was a web survey. The total sample size was 3,042 people.	Depression was measured using the PHQ-2. Resilience was measured with a website questionnaires developed by the authors of the article.	Age, gender, race, education, income, occupation, marital status, country of residence, number of people in the household, and date the survey was taken.	The study concluded that with every 1 SD increase in psychological resilience scores, there was a 69.3% decrease in the possibility of depression (OR = 0.31 [95%CI 0.252-0.383], p < 0.0001).	5/7	Yes	
**Author, year, country**	**Aim**	**Sample characteristics**	**Assessments**	**Confounding factors controlled**	**Follow-up duration**	**Main results**	**Quality**	**Is resilience a protective factor against mood disorders?**
Cohort studies								
Wu et al.,^29^ 2017, China	To examine the longitudinal effects of psychological resilience on childhood depression in a sample of left-behind children	The sample consisted of 386 left-behind children. The mean age and range were 12.2 years (8-17).	Depression was measured using the CDI. Resilience was measured using the Self-rating Scale of Psychological Resilience.	Age, sex, and baseline depressive symptoms.	A follow-up survey was completed a year later.	Higher psychological resilience was a significant protective factor against developing depression among left-behind children (OR: 0.96 [95%CI 0.94-0.99], p = 0.001).	8/10	Yes
Hiyoshi et al.,^30^ 2017, Sweden	To examine if physical and psychological characteristics in late adolescence, including factors previously linked with BD (BDI, asthma, and allergy), are associated with subsequent BD in adulthood	The sample consisted of 213,693 men born between 1952 and 1956 who participated in compulsory military conscription assessments in late adolescence. These assessments happened between the ages of 17 and 20. Total cohort n = 213,693: BD: n = 1,495; depression: n = 7,106	BD and depression were measured using the ICD-8. Resilience was measured using a semi-structured interview with a psychologist.	Age, sex, BMI, asthma, allergies, grip strength, cognitive ability, height, erythrocyte sedimentation rate, disease at conscription, region of residence, household crowding, and socioeconomic index in 1960.	Follow-up started immediately after the conscription assessment and ended on the date of the first diagnosis of BD (or anxiety or depression), death, emigration, or 31 December, 2009, whichever occurred first.	Higher stress resilience was associated with a lower risk of BD and depression	9/10	Yes
Hoorelbekeet al.,^31^ 2019, Belgium	To test the role of positive affect as a central resilience factor following remission from depression	85 patients were examined in a 7-day intervention that explored the interplay between five transdiagnostic vulnerabilities and protective factors in daily life.	Depression was measured at baseline using the BDI-II-NL. Resilience was measured through self-report questionnaires associated with the intervention.	N/A	The follow-up period lasted for 7 days.	The findings suggested a central role for positive affectivity as a key resilience factor because it positively impacted the cognitive risk and protective factors over time in RMD patients.	3/10	Yes
Navrady et al.,^32^ 2017, Scotland	To examine whether increased neuroticism and reduced resilience are downstream mediators of genetic risk for depression and whether they contribute independently to risk	Participants were sampled from the Generation Scotland: Scottish Family Health Study. At baseline, 664 individuals met the criteria for clinical MDD (16%) and 3,502 were non-MDD cases (84%). A total of 1,068 individuals in the mental health follow-up sample met the criteria for self-report MDD (26%), with 3098 classified as non-MDD cases (74%).	Participants were screened for a clinical diagnosis of MDD at baseline using the SCID-I. During re-contact, self-report MDD was measured using the CIDI-SF. Resilience was measured using the Brief RS.	Age at re-contact.	The baseline screening took place between 2006 and 2011. In 2014, participants were contacted and invited to participate in a follow-up assessment	Resilience protected against MDD. OR (SCID): 0.44, (95%CI 0.40, 0.48), p < 0.001 OR (CIDI-SF): 0.43, (95%CI 0.40, 0.47), p < 0.001.	8/10	Yes
Interventional studies								
Konradt et al.,^33^ 2018, Brazil	To assess the effects of resilience on the severity of depressive and anxious symptoms after brief cognitive psychotherapy for depression	91 drug-free adults (18-29 years old) with MDD were included in this study. 68 patients completed the study and were assessed post-intervention. 61 patients were assessed at a 6-month follow-up.	MDD diagnosis was measured using the SCID. The severity of depressive symptoms was measured using the HDRS. Resilience was measured using the 25-item RS.	N/A	Patients were assessed at baseline, post-intervention, and at six-month follow-up.	The resilience scores at post-intervention (125.2 ± 24.2) and at six-month follow-up (128 ± 28.53) were significantly higher than at baseline (105.5 ± 22.47, p < 0.001). Also, higher baseline resilience indicated lower depressive symptoms later on.	11/11	Yes
Seo et al.,^34^ 2017, South Korea	To examine whether basic military training can strengthen resilience in males with probable bipolar depression and probable unipolar depression	All participants were men. PUD: n = 66 PBD: n = 66 Controls: n = 66	The MDQ scale was used to screen for bipolar depression. The CES-D scale was used to screen for unipolar depression. The CD-RISC was used to measure resilience.	The control and mood disorder groups were matched for age, educational level, and BIS-11-R scores.	Follow up after 5 weeks of basic military training	There was no difference between the mood disorder group and the control group at baseline for resilience and the intervention did not change psychological resilience scores over 5 weeks.	7/9	No

BD = bipolar disorder; BDI = Beck Depression Inventory; BIS-11-R = Barratt Impulsiveness Scale-11-Revised; BMI = body mass index; CDI = Children’s Depression Inventory; CD-RISC = Connor-Davidson Resilience Scale; CES-D = Center for Epidemiologic Studies Depression Scale; CIDI-SF = Composite International Diagnostic Interview– Short Form; COVID-19 = coronavirus disease 2019; DSM = Diagnostic and Statistical Manual of Mental Disorders; HANDS = Harvard National Depression Screening Scale; HDRS = Hamilton Depression Rating Scale; HSC-25 = Hopkins Symptom Checklist-25; ICD = International Classification of Diseases; MADRS = Montgomery-Asberg Depression Rating; MDD = major depressive disorder; MDQ = mood disorders questionnaire; MINI = Mini-International Neuropsychiatric Interview; N/A = not available; NOS = not otherwise specified; NSCH = National Survey of Children’s Health; PBD = probable bipolar depression; PHQ-2 = Patient Health Questionnaire-2; PUD = probable unipolar depression; QoL = quality of life; RS = Resilience Scale; RSA = Resilience Scale for Adults; SCID = Structured Clinical Interview for DSM-IV; SCL-90-R = Symptom Checklist 90-R; OR = odds ratio; SD = standard deviation.

### Psychological resilience and mood disorders: evidence from cross-sectional studies

Seventeen cross-sectional studies compared psychological resilience between individuals with mood disorders (depression or BD) and individuals without mood disorders. The studies assessed a diverse population, including pregnant women, children, adults, and individuals facing stressful/traumatic situations. All 17 studies found that individuals with mood disorders were less resilient than individuals without mood disorders.

#### Psychological resilience and mood disorders during pregnancy

Zhang et al.^13^ examined the prevalence of prenatal depression and explored its associated factors. Their study included 605 pregnant women divided into women with prenatal depression (n *=* 172) and women with no prenatal depression (n *=* 433). Depression was assessed using the Center for Epidemiologic Studies Depression Scale (CES-D) self-report instrument. The study found that women with prenatal depression had lower psychological resilience scores than women without prenatal depression.

#### Psychological resilience and mood disorders in children

Elmore et al.^14^ examined the association between adverse childhood experiences and positive childhood experiences on the outcome of depression. The study included 40,302 children 8 years or older who were divided into a currently depressed group (n = 2,174) and a not currently depressed group (n = 38,128). Depression was assessed using a self-report assessment, the National Survey of Children’s Health (NSCH). The study found that child psychological resilience reduced the odds of depression four-fold and children who were currently depressed were less likely to report child psychological resilience.

#### Psychological resilience and mood disorders in adults

Seok et al.^15^ examined the relationship between early-life stress, depression tendency, and psychological resilience in individuals with MDD. The sample included 52 individuals divided into a group with MDD (n = 26) and a group without MDD (n = 26). Depression was assessed using the Korean version of the Structured Clinical Interview for DSM-IV (SCID). The study concluded that psychological resilience scores were lower for the group with MDD than for the group without MDD. Cha et al.^16^ examined the demographic and clinical factors related to psychological resilience in euthymic patients with BD. The sample included 124 individuals divided into a group with BD (n = 62) and a group without BD (n = 62). BD was diagnosed according to the DSM-IV-TR criteria. The study concluded that psychological resilience scores were lower in the group with BD than in the group without BD. Ozawa et al.^17^ examined the degree and quality of psychological resilience in patients with depression in the context of remission status, spirituality/religiosity, and family members’ psychological resilience levels, which had never been investigated prior to this study. The sample included 136 individuals divided into individuals without depression (n *=* 36) and individuals with depression (n *=* 100). Depression was assessed using the International Classification of Diseases (ICD) criteria. The study concluded that psychological resilience scores were lower in the depressed group compared to the control group. Deng et al.^18^ examined the relationship between psychological resilience and cognitive functioning in individuals with schizophrenia and BD. The sample included 167 individuals divided into a group with schizophrenia (n = 81), a group with BD (n = 34), and a group with no mood disorders (n = 52). Mood disorders were diagnosed with a clinical interview. The study concluded that psychological resilience scores were lower in groups with schizophrenia and BD compared to the control group. Bozikas et al.^19^ examined the association between resilience and social functioning in patients with BD. A sample of 80 individuals was divided into a group with BD (n = 40) and a group without BD (n = 40). BD was diagnosed according to the DSM-IV, and the diagnosis was confirmed using the Greek version of the Mini-International Neuropsychiatric Interview (MINI). The study concluded that psychological resilience scores were lower in the group with BD than in the control group. Post et al.^20^ examined the impact of psychological resilience, internalized stigma, and psychopathology on QoL.^20^ The sample included 137 individuals divided into a group with BD (n *=* 60) and a group without BD (n *=* 77). BD was diagnosed following the DSM-IV-TR criteria. The study concluded that psychological resilience scores were lower in the group with BD compared to the group with no BD. Vieira et al.^8^ examined the mediation effect of psychological resilience on the relationship between childhood trauma and mood disorders. The sample included 1,244 individuals who were divided into a group with MDD (n *=* 317), a group with BD (n *=* 90), and a group with no mood disorders (n *=* 837). Mood disorders were assessed using the MINI-PLUS. The study concluded that psychological resilience scores were lower in mood disorder groups than in the control group. Uygun et al.^21^ examined the association between psychological resilience and disease onset, QoL, and prognosis of BD in euthymic patients. The sample included 120 individuals divided into a group with BD (n *=* 90) and a group without BD (n *=* 30). BD was diagnosed using a clinical interview. The study concluded that psychological resilience scores were lower in the group with BD compared to the group without BD.

#### Psychological resilience and mood disorders during stressful/traumatic situations

Aroian et al.^22^ examined the relationship between psychological resilience, demographic characteristics, immigration demands, and depression in a sample of 450 adult Russian immigrants to Israel between 1990 and 1995. The sample was divided into a group with depression (n *=* 241) and a group without depression (n *=* 209). Depression was assessed using a 13-item self-report Depression Scale (Russian language version of the Symptom Checklist 90-R [SCL-90-R]). The study concluded that individuals with high psychological resilience scores had a more than two-fold greater likelihood of not being depressed compared to individuals with a low psychological resilience score. Hsieh et al.^23^ examined the relationship among recent workplace violence, depressive tendency, social support, and psychological resilience of victimized nurses. The sample was recruited from two hospitals in Taiwan. One hundred fifty-nine nurses met the inclusion criteria and were divided into a group with a depressive tendency (n *=* 74) and a group without a depressive tendency (n *=* 85). Depression was assessed using the self-report instrument CES-D, with a cut-off of 14 for depressive tendency. The study findings concluded that the group with a depressive tendency was significantly less resilient than the group without a depressive tendency. Blackmon et al.^24^ examined the relationships between depression, psychological resilience, and other sociodemographic factors of individuals highly exposed to Hurricane Katrina in 2005 and the Deepwater Horizon Oil Spill in 2010. The sample included 294 Mississippi Gulf Coast residents living near the Gulf of Mexico and was divided into a group with depression and a group without depression. Twenty-one percent of the sample had depression. Depression was assessed using the self-report CES-D, with a cut-off for depression of 16. The study concluded the individuals with depression were significantly less resilient than individuals without depression. Simpkin et al.^25^ examined how stress from uncertainty relates to psychological resilience among pediatric residents and whether these attributes are associated with depression and burnout. The sample included 86 residents and depression was assessed using the self-report instrument Harvard National Depression Screening Scale (HANDS). The study concluded that the pediatric residents with depression were significantly less resilient than the pediatric residents without depression. Poudel-Tandukar et al.^26^ examined the association between psychological resilience and anxiety or depression in Bhutanese adults resettled in Western Massachusetts. The sample included 450 Bhutanese refugees aged 20-65 and residing in Massachusetts divided into refugees with depression (n *=* 54) and refugees without depression (n *=* 171). Depression was assessed using the Hopkins Symptom Checklist-25 (HSC-25) self-report scale with a mean cut-off of ≥ 1.75 for moderate to severe symptoms. The study concluded that refugees in the highest tertile by psychological resilience scores had a significantly decreased risk of depression. Yörük et al.^27^ examined the relationship between psychological resilience, burnout, stress, and sociodemographic factors with depression in nurses and midwives during the coronavirus disease 2019 (COVID-19) pandemic. The sample included 377 midwives and nurses and was divided into a group with depression (n *=* 120) and a group without depression (n *=* 257). Depression was assessed using the self-report Beck Depression Inventory (BDI), with a cut-off of 17 for depression. The study concluded that the midwives and nurses with depression were significantly less resilient than the midwives and nurses without depression. Barzilay et al.^28^ examined the role of psychological resilience for healthcare workers during the COVID-19 pandemic. The total sample size was 3,042 people and depression was assessed using the Patient Health Questionnaire-2 (PHQ-2) self-report scale. The study concluded that with every 1 SD increase in psychological resilience scores, there was a 69.3% decrease in the possibility of depression.

#### Psychological resilience and mood disorders: evidence from the meta-analysis of the cross-sectional studies

**Figure 2:**
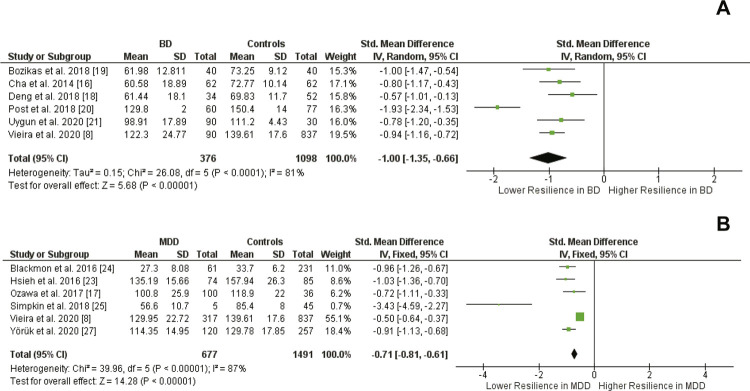
Meta-analysis comparing the psychological resilience scores between individuals with bipolar disorder (BD) and controls (A) and individuals with depression and controls (B). SD = standard deviation; IV= interval variable; 95% CI = 95% confidence interval; MDD = major depressive disorder.

Our meta-analysis showed that individuals with BD presented significantly lower levels of psychological resilience compared to controls (SDM: -1.00 [95%CI -1.35 to -0.66], p *<* 0.001) (Figure 2A). In addition, individuals with depression had significantly lower levels of psychological resilience compared to controls (SDM: -0.98 [95%CI -1.31 to -0.64], p *<* 0.001) (Figure 2B).

#### Quality assessment for cross-sectional studies

The quality of all 17 cross-sectional studies was assessed using JBI Systematic Review’s Checklist for Analytical Cross-Sectional Studies. However, we decided to omit question 4 because we were not assessing any specific condition. Hence, each article was scored out of a maximum possible score of 7. Our assessment showed that the total scores ranged from 4 to 7. The mean score for all 17 articles was 5.8 (Table 1).

## Psychological resilience and mood disorders: evidence from longitudinal studies

Four cohort studies were included in the systematic review. All the studies showed that psychological resilience protects against the development of mood disorders.

Wu et al.^29^ examined the longitudinal effects of psychological resilience on depression in a Chinese sample of left-behind children. The prevalence rates of depression at baseline and 1-year follow-up were 12.7 and 8.5%, respectively. The study found that children with higher baseline psychological resilience (adjusted odds ratio [OR] = 0.97; 95%CI 0.95-0.99) were at a reduced risk for developing depression at the 1-year follow-up, adjusting for age, sex, and baseline depressive symptoms. Hiyoshi et al.^30^ examined whether physical and psychological characteristics in late adolescence were associated with subsequent BD in adulthood. A total of 213,693 men born between 1952 and 1956 who participated in compulsory military conscription assessments in late adolescence were followed up to 2009, excluding men with any psychiatric diagnoses at baseline. Psychological resilience was measured using a semi-structured interview with a psychologist and was stratified into “low,” “medium,” and “high” psychological resilience. High resilience was protective against depression (adjusted OR = 0.61; 95%CI 0.56-0.66) and BD (adjusted OR = 0.83; 95%CI 0.70-0.98). The study adjusted for age, sex, body mass index, asthma, allergies, grip strength, cognitive ability, height, erythrocyte sedimentation rate, disease at conscription, region of residence, household crowding, and socioeconomic index in 1960. Hoorelbeke et al.^31^ examined the cognitive risk and protective factors following remission from depression. The study utilized a 7-day experience sampling period in 85 patients with remitted depression and examined the interplay between five transdiagnostic vulnerabilities and protective factors (including psychological resilience) in daily life. The study suggests a significant role for positive affectivity as a key resilience factor. It positively impacted cognitive risk and protective factors over time in remitted patients with depression. Navrady et al.^32^ assessed the moderating and mediating relationships between depression, polygenic risk score (PRS), neuroticism, resilience, and clinical and self-report depression in a large, population-based cohort. Participants were screened for a clinical diagnosis of MDD at baseline using the SCID-I. During the reassessment visit, self-report MDD was assessed using a questionnaire developed by the World Health Organization (WHO): The Composite International Diagnostic Interview-Short Form (CIDI-SF). A total of 1,068 individuals in the mental health follow-up sample met the criteria for self-report MDD (26%), with 3,098 classified as non-MDD cases (74%). A strong inverse relationship was found between resilience and clinically diagnosed depression (adjusted OR = 0.44; 95%CI 0.40-0.48). A similar relation was found between resilience and self-report MDD (adjusted OR = 0.43; 95%CI 0.40-0.47). These findings were adjusted for age, sex, and PRS.

### Quality assessment for longitudinal studies

The quality of all four cohort studies was assessed using JBI Systematic Review’s Checklist for Cohort Studies. However, from the checklist, we decided to omit question 6 because our methodology did not necessarily require the subjects to be free of the outcome at the baseline. Hence, each article had a maximum possible score of 10. The total scores ranged from 3 to 9. The mean score for all four articles was 7 (Table 1).

## Psychological resilience and mood disorders: evidence from interventional studies

Two interventional studies were included in the systematic review. One of the two studies (50%) found that the intervention increased the psychological resilience score and found that higher baseline psychological resilience indicated lower depressive symptoms at follow-up among individuals diagnosed with MDD. Konradt et al.^33^ conducted a randomized clinical trial including 91 young adults diagnosed with MDD and assessed the effects of psychological resilience on the severity of depressive symptoms after brief cognitive psychotherapy interventions (Cognitive Behavior Therapy [CBT] or Narrative Cognitive Therapy [NCT]) for depression. The study found a higher psychological resilience at post-intervention and at 6-month follow-up. Moreover, higher baseline psychological resilience indicated lower depressive symptoms at post-intervention and at 6-month follow-up. Seo et al.^34^ conducted a quasi-experimental study and examined whether basic military training can strengthen psychological resilience in males with probable bipolar depression (PBD) and probable unipolar depression (PUD). The study population consisted of Korean conscripts admitted to a basic military training camp in 2015. All participants were men. There were 66 participants in the PUD group, 66 in the PBD group, and 66 in the control group. There were no differences in psychological resilience between the mood disorder groups and the control group at baseline and the intervention did not change resilience scores over 5 weeks. These findings can probably be explained by the short follow-up period (5 weeks).

### Quality assessment for interventional studies

The quality of the RCT study was assessed using JBI Systematic Review’s Checklist for Randomized Controlled Trials. However, we decided to omit questions 4 and 5. Question 4 was omitted because it was not possible to blind participants to the treatment with psychotherapy. Similarly, question 5 was omitted because it was not possible to blind those delivering treatment. Hence, the maximum possible score was 11. The RCT included in this systematic review had a score of 11 (Table 1).

The quality of the quasi-experimental study was assessed using JBI Systematic Review’s Checklist for Quasi-Experimental studies. The maximum possible score was 9. The quasi-experimental study included in this systematic review had a score of 7 (Table 1).

## Discussion

Our meta-analysis of the cross-sectional data showed that individuals suffering from mood disorders had lower psychological resilience scores than individuals without mood disorders. Moreover, results from our systematic review showed evidence from longitudinal studies suggested that higher psychological resilience protected against the development of mood disorders. Lastly, few interventional studies indicated that psychotherapy interventions may improve psychological resilience. One interventional study also showed that higher baseline psychological resilience indicated lower depressive symptoms at follow-up in individuals with MDD.

Psychological resilience is the ability to effectively cope with the stressors of life to maintain good mental health.^10^ Twenty-two of the 23 (95.7%) studies included in the present systematic review concluded either that individuals suffering from mood disorders had lower psychological resilience scores than individuals without mood disorders or that psychological resilience protected against the development of mood disorders. These conclusions are in line with two other systematic reviews in the field demonstrating that psychological resilience positively impacts the mental health of individuals.^11,12^ However, it is important to highlight that those reviews were focused on specific populations (individuals forced to migrate and individuals with somatic illness) and they did not specifically assess the impact of psychological resilience on mood disorders.

There is no current gold standard assessment to measure psychological resilience. However, Windle et al.^35^ systematically reviewed the psychometric rigor of resilience measurement scales developed for use in general and clinical populations. In the review, the CD-RISC, the Resilience Scale for Adults (RSA), and the Brief Resilience Scale (BRS) received the best psychometric ratings. In this sense, it is important to highlight that 12/23 (52%) studies included in our systematic review used one of the three aforementioned resilience scales.

It is known that mood disorders have multifactorial etiology. For instance, a recent study showed that childhood trauma partly mediated the impact of family history on mood disorder diagnosis in adulthood, which suggests that childhood trauma might act as an environmental trigger that, by interacting with a vulnerable genetic background, can lead to the onset of mood disorders.^36^ Psychological resilience has also been found to moderate the relationship between stress and childhood depression,^37^ indicating that individuals who suffered from stress but were more resilient were less likely to develop depression. The same findings were replicated by Vieira et al.,^8^ who showed that psychological resilience mediated the relationship between childhood trauma and mood disorders in young adults. These data reinforce the importance of investigating psychological resilience in the context of mood disorders.

Importantly, interventions such as mindfulness show promise for increasing psychological resilience. Galante et al.^38^ conducted an RCT to assess whether mindfulness courses for university students would improve their resilience to stress. Their findings suggest that mindfulness courses effectively increased resilience to stress in university students. Moreover, a recent systematic review found that interventions based on a combination of CBT and mindfulness techniques appear to impact individual resilience positively.^39^ We believe more research into mindfulness techniques and interventions can establish a more concrete understanding of the relationship between psychological resilience and mood disorders.

Our findings should be interpreted considering some limitations. First, the systematic review only included two interventional studies, which had conflicting results. Hence, looking at more interventional studies would have strengthened the conclusions based on interventions. Second, only four longitudinal studies were included and level of evidence for the causal relationship between psychological resilience and mood disorders is still weak. Finally, a meta-analysis of interventional and longitudinal studies was not performed because of the heterogeneity of the studies included. Despite these limitations, our systematic review incorporated a diverse population, including children and adults who experienced several types of stressful situations (ex.: childhood trauma, immigration, pregnancy, dealing with the COVID-19 pandemic, etc.). This allowed us to describe the impact of psychological resilience in mood disorders in the context of various stressful situations individuals may face.

## Conclusion

To the best of our knowledge, this systematic review is the first in its field to look at the relationship between resilience and mood disorders through various circumstances endured by the individuals. Our results showed that individuals suffering from mood disorders had lower psychological resilience scores than individuals without mood disorders. In addition, higher psychological resilience scores may lead to reduced rates of mood disorders in the context of many adverse situations. In terms of future research into the impact of psychological resilience on mood disorders, we recommend more longitudinal studies to establish a causal relationship between psychological resilience and mood disorders. Also, more research is needed on interventions that can positively impact individuals with mood disorders.
